# Chronic semaglutide treatment attenuates cue-induced reinstatement of cocaine-seeking and cocaine-induced stereotypy in male and female rats

**DOI:** 10.64898/2026.09.21.753157

**Published:** 2026-09-28

**Authors:** Christopher A. Turner, Natalia Morales Pagán, Daniela Pereira, Eileen Y. Sun, Rachel E. Himelhoch, Stephen E. Chang, Shelly B. Flagel

**Affiliations:** 1 Michigan Neuroscience Institute, University of Michigan, Ann Arbor, Michigan, USA, 48109; 2 Neuroscience Graduate Program, University of Michigan, Ann Arbor, Michigan, USA, 48109; 3 Department of Psychology, College of Literature, Science, and the Arts, University of Michigan, Ann Arbor, Michigan, 48109, USA; 4 Department of Psychiatry, University of Michigan, Ann Arbor, Michigan, USA, 48109

## Abstract

Following cocaine self-administration, male and female rats received daily semaglutide during a 30-day abstinence period before cue- and cocaine-induced reinstatement tests. Semaglutide attenuated cue-induced reinstatement and cocaine-induced stereotypy, although operant responding was elevated following cocaine priming. These findings support further investigation of semaglutide as a treatment for cocaine use disorder.

## Introduction

Glucagon-like peptide-1 (GLP-1) receptor agonists, such as semaglutide, are best known for reducing food intake and sustaining weight loss [1]. These medications also reduce alcohol craving and consumption [2], and emerging evidence suggests they reduce the risk of substance use disorders, including cocaine use disorder [3]. Consistent with these findings, preclinical studies show that acute GLP-1 receptor agonist treatment reduces cocaine-taking and -seeking [4,5]. However, repeated treatment may better model the prolonged exposure associated with clinical use.

A recent study found that repeated semaglutide shifted reward preference in rats from cocaine toward food under concurrent access [6], but whether chronic semaglutide attenuates relapse-relevant behaviors, including cue- and cocaine-induced reinstatement, remains unknown. We therefore assessed the effects of chronic semaglutide treatment, initiated during abstinence after cocaine self-administration, on cue- and cocaine-induced reinstatement in rats.

## Materials & Methods

Subjects were male and female Sprague-Dawley rats weighing 250–325 g upon arrival. All procedures conformed to the *Guide for the Care and Use of Laboratory Animals* (National Research Council, 2011) and were approved by the University of Michigan Institutional Animal Care and Use Committee. Figure 1A shows the timeline; details are in Supplementary Information.

### Cocaine Self-administration

Rats self-administered cocaine through indwelling jugular catheters. An active-port response produced an intravenous cocaine infusion (0.875 mg/kg/infusion, 50 μL over 2.8 s) paired with a 20-s port-light cue, and inactive-port responses were without consequence. To standardize the number of cocaine-cue pairings, infusion criteria (IC) limited infusions across 14 daily sessions: 3 sessions at IC5, 4 at IC10, 3 at IC20, and 4 at IC45. During IC45, cocaine was reduced to 0.35 mg/kg/infusion to elevate operant responding.

### Abstinence and Semaglutide Treatment

Daily subcutaneous vehicle (VEH) or semaglutide (SEMA) injections began on abstinence day 5, and continued throughout abstinence, extinction, and testing, resulting in at least 30 days of treatment before cue-induced reinstatement. SEMA dosing began at 7 μg/kg and increased by 7 μg/kg daily to a maintenance dose of 70 μg/kg [7].

### Extinction

Following 30 days of abstinence, rats returned to the self-administration chambers for 45-min extinction sessions during which responses in either port had no consequence. Training continued until rats made ≤12 active-port responses during each of two consecutive sessions. Rats failing to meet this criterion before reinstatement testing were excluded from corresponding analyses.

### Cue-induced Reinstatement

Cue-induced reinstatement was tested ≈1 h after the last extinction session. During the 45-min test, active-port responses presented the cocaine-associated cue light but no cocaine.

### Cocaine-induced Reinstatement

After additional extinction, cocaine-induced reinstatement was examined 24 h later using a within-session ascending dose-response procedure. Rats received cocaine (0, 5, 10, 15 mg/kg, i.p.) before successive 45-min blocks over ≈4 h. Behavior was video-recorded and scored manually and with LabGym, a behavior-analysis platform [8].

### Statistical Analyses

Statistical details are in the Supplementary Information. Sex differences were observed only in body weight; results for other outcomes are reported across sexes.

## Results

Sample sizes for acquisition, body weight, and extinction were VEH: n=27 (15F/12M) and SEMA: n=26 (14F/12M). After attrition, cue-induced reinstatement sample sizes were VEH: n=21 (10F/11M) and SEMA: n=23 (14F/9M). Cocaine-induced reinstatement was limited to cohorts 2 and 3, with sample sizes of VEH: n=18 (11F/7M) and SEMA: n=17 (10F/7M).

### Cocaine self-administration acquisition

Acquisition of cocaine self-administration was indicated by increased responding across training (effect of IC, F_3,61.445_ = 246.434, *p* < 0.001) and greater active- than inactive-port responding (effect of Port, F_1,134.154_ = 2528.516, *p* < 0.001; IC x Port interaction, F_3, 86.582_ = 660.109, *p* < 0.001; Fig. 1B). Treatment groups were counterbalanced using acquisition measures.

### Chronic semaglutide reduced body weight

Chronic semaglutide reduced body weight in both sexes (effect of Treatment, F_1, 49_ = 223.11, *p* < 0.001; Males: t(22) = 11.805, *p* < 0.001; Females: t(27) = 9.512, *p* < 0.001; Fig. 1C). To determine whether weight differences contributed to reinstatement performance, we correlated body weight with responding. Body weight at the time of testing was not correlated with cue- or cocaine-induced responding (data not shown).

### Chronic semaglutide did not affect the extinction of cocaine-seeking

Semaglutide did not affect extinction before cue-induced (Fig. 1D) or cocaine-induced reinstatement (Fig. 1H), even when the first 5 min of initial extinction were analyzed separately (data not shown).

### Chronic semaglutide attenuated cue-induced reinstatement of cocaine-seeking

During cue-induced reinstatement, both treatment groups responded more in the active than inactive port (effect of Port, Wald χ^2^(1) = 55.14, *p* < 0.001; Fig. 1E). However, VEH-treated rats showed a 9-fold difference between active and inactive responding, whereas SEMA-treated rats exhibited a 2.7-fold difference (Treatment x Port interaction, Wald χ^2^(1) = 7.99, *p* = 0.005; Fig. 1E).

To assess responding above the extinction baseline, active-inactive difference scores were compared between the last extinction session preceding cue-induced reinstatement and the test session (Figs. 1F, 1G). Only VEH-treated rats showed cue-induced reinstatement (Treatment x Session interaction, F_1, 42_ = 4.668, *p* = 0.036; Fig. 1F), as indicated by a significant increase in the active-inactive difference score from extinction to reinstatement in VEH- (t(20) *=* 3.205, p = 0.007) but not SEMA-treated rats (Fig. 1F). In agreement, during reinstatement, the active-inactive response difference was greater in VEH- than SEMA-treated rats (Welch’s t-test: t(31.863) = 2.094, *p* = 0.049; Fig. 1G).

These effects were unlikely to be attributable to nonspecific suppression of activity because inactive-port responding during the cue-induced reinstatement test did not significantly differ between treatment groups (t(42) = 1.363, *p* = 0.180; Fig. 1E), and baseline locomotor activity prior to cocaine-induced reinstatement was comparable between groups (t(33) = 1.354, *p* = 0.185; data not shown).

### Cocaine-induced reinstatement of cocaine-seeking

Rats responded more in the active than inactive port during cocaine-induced reinstatement (effect of Port, F_1, 264_ = 167.812, *p* < 0.001; Fig. 1I). Active-port responding was significantly greater after 5 and 10 mg/kg cocaine than after 0 mg/kg, but decreased at 15 mg/kg, generating an inverted-U dose-response curve (effect of Dose, F_3, 264_ = 27.526, *p* < 0.001; Dose x Port interaction, F_3, 264_ = 11.058, *p* < 0.001; Fig. 1I). Across doses, responding differed between VEH- and SEMA-treated rats (Treatment x Dose interaction, F_3, 264_ = 3.637, *p* = 0.013), regardless of port (Fig. 1I). At 15 mg/kg, SEMA-treated rats responded more than VEH-treated rats (*p* = 0.026; Fig. 1I); however, the Treatment x Port interaction was not significant, indicating increased overall responding rather than selective active-port responding.

### Chronic semaglutide attenuated cocaine-induced stereotypy

Video recordings following the 15 mg/kg dose were scored manually and using LabGym [8]. Manual scoring focused on stereotypy, characterized by repetitive movements such as head swaying, commonly observed following repeated psychostimulant exposure [9]. Both methods showed that chronic semaglutide significantly attenuated the percentage of time spent displaying stereotypy (Handscore: U = 86, *p* = 0.027; LabGym: U = 88, *p* = 0.032; Fig. 1J). Figure 1K shows a plot of LabGym classifications. Relative to VEH-treated rats, SEMA-treated rats spent significantly more time locomoting (t(33) = 2.984, *p* = 0.005) and rotating (Welch’s t-test: t(22.975) = 2.603, *p* = 0.016; Fig. 1L), suggesting that semaglutide shifted the behavioral response to high-dose cocaine from stereotypy toward locomotor activity.

## Discussion

Chronic semaglutide attenuated cue-induced reinstatement of cocaine-seeking and cocaine-induced stereotypy. The incentive-sensitization theory of addiction proposes that repeated drug use induces neural adaptations that sensitize the brain to both the direct effects of the drug (psychomotor sensitization) and drug-associated cues (incentive sensitization), allowing subsequent exposures to trigger pathological drug-seeking [10,11]. Our findings suggest that semaglutide attenuates the expression of incentive and psychomotor sensitization, although further investigation is needed to confirm this interpretation. Additionally, modest cue-induced responding warrants replication under conditions producing more robust reinstatement [12].

SEMA-treated rats showed greater operant responding at the highest cocaine dose during cocaine-induced reinstatement. This likely reflects reduced competition from stereotypy: SEMA-treated rats exhibited greater locomotion and were able to respond, whereas stereotypy interfered with responding in VEH-treated rats. Rather than indicating enhanced cocaine seeking, the increase in responding may reflect a semaglutide-induced shift in the behavioral response to cocaine from stereotypy toward locomotion. Thus, both findings are consistent with attenuated responses to cocaine and cocaine-associated cues. Because responding during reinstatement did not result in cocaine delivery, whether attenuated cocaine effects promote compensatory intake requires further testing.

These findings are consistent with reports that acute GLP-1 receptor agonist treatment attenuates cue- and cocaine-primed reinstatement [4,5]. Notably, attenuated cue-triggered cocaine seeking contrasts with our previously reported enhancement of responding for a food reward and associated cue following chronic semaglutide [13], suggesting reward-specific effects on incentive motivation. Consistent with this interpretation, Heslep et al. found that repeated semaglutide affected cocaine reinforcement more than food reinforcement [6]. These effects may reflect interactions between cocaine-induced adaptations in mesolimbic dopamine signaling [14] and GLP-1 receptor agonist modulation of this system [15], potentially altering psychomotor sensitization to cocaine and the incentive salience of cocaine-associated cues while differentially affecting motivation for food.

In summary, chronic semaglutide attenuated cue-induced reinstatement of cocaine-seeking and cocaine-induced stereotypy, potentially reflecting reduced incentive and psychomotor sensitization, respectively. Semaglutide may offer a novel strategy for reducing relapse vulnerability in cocaine use disorder, warranting further investigation.

## Figures and Tables

**Figure 1.**
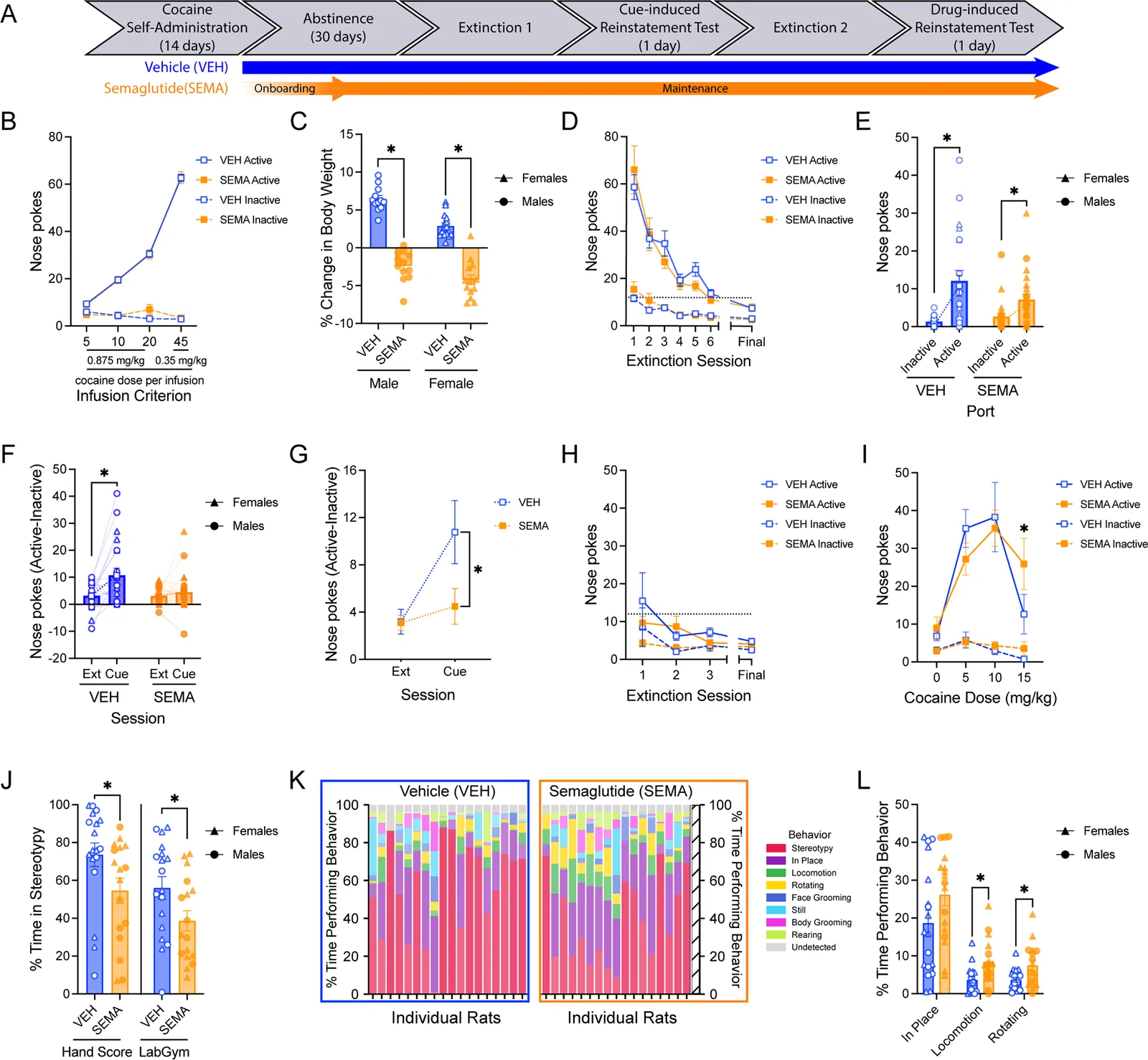
Unless noted, line graphs show group means ± SEM of active-port (solid line) and inactive-port (dashed line) nose pokes, and bars depict group means ± SEM with individual data points shown. Females are indicated by triangles and males by circles. (A) Experimental timeline: vehicle (VEH) or semaglutide (SEMA) treatment began on abstinence day 5 (dose-escalated from 7 μg/kg to a maintenance dose of 70 μg/kg) and continued for the duration of the study. (B) Nose pokes during acquisition of cocaine self-administration across infusion criteria (IC). Rats responded more in the active port as IC increased (*p* < 0.001). (C) Percent change in body weight from baseline after 30-days of SEMA or VEH treatment. SEMA-treated males (*p < 0.001) and females (*p < 0.001) showed a greater reduction in body weight compared to their VEH-treated counterparts. (D) Nose pokes across the extinction sessions preceding cue-induced reinstatement. Drug-seeking behavior decreased over time (*p* < 0.001). (E) Nose pokes made during the cue-induced reinstatement test. Dashed lines indicate the active/inactive response ratio. Both groups showed greater responding in the active port than in the inactive port (*p < 0.001). (F) Active-inactive difference scores for the final extinction session versus the cue-induced reinstatement test. Only VEH-treated animals showed a significant increase in their difference scores during the cue-test relative to extinction (*p = 0.007). (G) During cue-induced reinstatement, VEH-treated rats had a significantly greater active-inactive difference score than SEMA-treated rats (*p = 0.049). (H) Nose pokes across the extinction sessions preceding cocaine-induced reinstatement. Overall responding decreased over time (*p* = 0.007). (I) Nose pokes across the cocaine dose-response curve for cocaine-induced reinstatement. At 15 mg/kg, SEMA-treated rats responded significantly more than VEH-treated rats (*p = 0.026). (J) Percent time in stereotypy following 15 mg/kg cocaine, scored manually (left) and via LabGym (right). Both methods showed that SEMA attenuated stereotypy (HandScore: *p = 0.027; LabGym: *p = 0.032). (K) Representative LabGym raster plot depicting percentage of time performing each of the eight classified behaviors (stereotypy, in-place, locomotion, rotating, face grooming, still, body grooming, and rearing; below-threshold classifications labeled undetected), averaged across nine 30-s subsamples per animal (columns); color intensity reflects classifier confidence. (L) Percent time spent performing the most frequently detected LabGym-classified behaviors at 15 mg/kg. Treatment groups differed significantly in locomotion (*p = 0.005) and rotating (*p = 0.016).
